# Effect of dehydration during pregnancy on birth weight and length in West Jakarta

**DOI:** 10.1017/jns.2021.59

**Published:** 2021-08-27

**Authors:** Erry Y. Mulyani, Hardinsyah, Dodik Briawan, Budi I. Santoso, Idrus Jus'at

**Affiliations:** 1 Department of Nutrition, Faculty of Health Sciences, Esa Unggul University, Jalan Arjuna Utara No.9, Kebon Jeruk11510, West Jakarta, Indonesia; 2 Department of Community Nutrition, Faculty of Human Ecology, Bogor Agricultural University, Bogor, Indonesia; 3 Department of Obstetrics and Gynaecology, Faculty of Medicine, University of Indonesia - Dr. Cipto Mangunkusumo General Hospital, Depok, Indonesia

**Keywords:** Dehydration, Fetal growth, Maternal nutrition, Pregnancy, Water intake

## Abstract

Nutrition and maternal behavior are critical factors in fetal development. Maternal water intake is necessary to regulate metabolism and may influence fetal growth. This study aims to determine the effect of dehydration during pregnancy on birth weight and length. This cohort-prospective study took place in the area of Kebon Jeruk District Health Centre. A total of 38 subjects of pregnant women in their second trimester were examined. Subject characteristics were collected through direct measurements and interviews. Urine and blood samples were collected at the sixth trimester (32–34 and 35–37 weeks) to determine hydration status. Nutritional status was collected through food recall, while birth weight and length were obtained from the anthropometric measurements 30 min after birth. From a total of 38 subjects, 20 were dehydrated, and 18 were well hydrated. There was a significant relationship between hydration status and water intake, birth weight and length, head circumference, and chest circumference. After being corrected to the level of water intake, the difference in birth weight and length between the two groups were 500⋅6 g and 0⋅4 cm, and 0⋅8 cm and 1⋅4 cm for the head circumference and chest circumference (*P* < 0⋅05). It is recommended for mothers to monitor their weight and ensure fluid intake of 3⋅0 l per day. Further research requires more subjects to observe the effects of chronic maternal dehydration on pregnancy output and a cohort study that monitors infant development in the first six months of life.

## Introduction

During pregnancy, nutrition and maternal behavior are essential in fetal brain development. Maternal physiological and metabolism adaptation throughout pregnancy would impact fetal development^(1, 2)^. Concurringly, several studies suggested that maternal diet affects pregnancy output^(3, 4)^. For example, Timmermans *et al.* (2011) mentioned that dietary patterns of grains and fruit or fat (processed meat diet) are associated with concentrated blood biomarkers influencing endothelial function (folate, homocysteine/tHcy, and high-sensitivity C-reactive protein/hs-CRP). Another study found that dietary intake and composition are a parameter of maternal and fetal well-being. Deficiencies in the consumption of such intakes; protein, vitamins, PUFAs can cause stunted fetal/offspring development while consuming certain foods can have a beneficial impact on fetal growth and development^(5)^. For example, lack of retinol intake in dietary composition impacts fetal growth, but not on fetal kidney growth in late pregnancy^(6)^. Another thing to consider is the fulfillment of nutritional intake during preconception^(7)^.

Usually, plasma volume increases progressively during pregnancy. Most of this 50 % increase occurs at 34 weeks gestation, proportional to the birth weight. Because the expansion in plasma volume is greater than the increase in red blood cell mass, there is a decrease in hemoglobin concentration, hematocrit, and red blood cell count^(8)^. Changes that occur during the pregnancy period cause an adjustment in water requirements during pregnancy. Water is an essential nutrient in metabolism, hydration, and health, especially in the body fluid balance mechanism^(9)^. Homeostasis is a system in which variables are regulated so that internal conditions remain stable and relatively constant^(10)^.

Hydration during pregnancy is requisite to sustain the balance of amniotic fluid, which is vital to maintain fetal well-being. Therefore, amniotic fluid deficiency (oligohydramnios) can have many effects on the prognosis of pregnancy^(11)^. Oligohydramnios complicates 3–5 % of all secondary pregnancies due to ruptured membranes, placental insufficiency, congenital anomalies such as bilateral renal agenesis, post-term pregnancy, twin-to-twin transfusions, fetal death, or idiopathic^(11–14)^.

Studies with pregnant rats and sheep showed that dehydration resulted in a low-birth-weight outcome, indicated by the presence of specific and hypertonic plasma hypernatremia and arterial hypertension^(15)^. Other studies on sheep explained that pregnant sheep with limited liquid intake were given intravenous fluid after delivery resulted in hypernatremia, hypertonic, and hypertension. The survived lamb was then infused with a hypotonic saline solution. A study on humans found that a mother with uterine hypertonicity and stress could influence her child's osmoregulation and systemic arterial hypertension^(16)^. Water is needed for body metabolism regulation, especially during pregnancy, considering its impacts on the fetus. However, research related to pregnancy hydration in humans is still scarce. Therefore, this study aims to determine how dehydration during pregnancy affects birth weight and length.

## Methods

### Design, place, and time

This cohort-prospective study aimed to investigate the effect of dehydration during pregnancy on birth weight and length. The study was conducted in seven areas of the Kebon Jeruk District Health Centre, namely Kebon Jeruk Health Centre, South Sukabumi Health Centre, North Sukabumi Health Centre, North Kedoya Health Centre, South Kedoya Health Centre, Duri Kepa Health Centre, and Kelapa Dua Health Centre in December 2016 to January 2018. This study was conducted according to the guidelines laid down in the Declaration of Helsinki, and all procedures involving human subjects were approved by the Health Research Ethics Commission of the Faculty of Medicine, University of Indonesia, Jakarta, in the form of an Ethical Approval Pass with Number: No.869/UN2.F1/ETHICS/2016 dated October 10, 2016. Written informed consent was obtained from all subjects.

### Subject

**Fig. 1.**
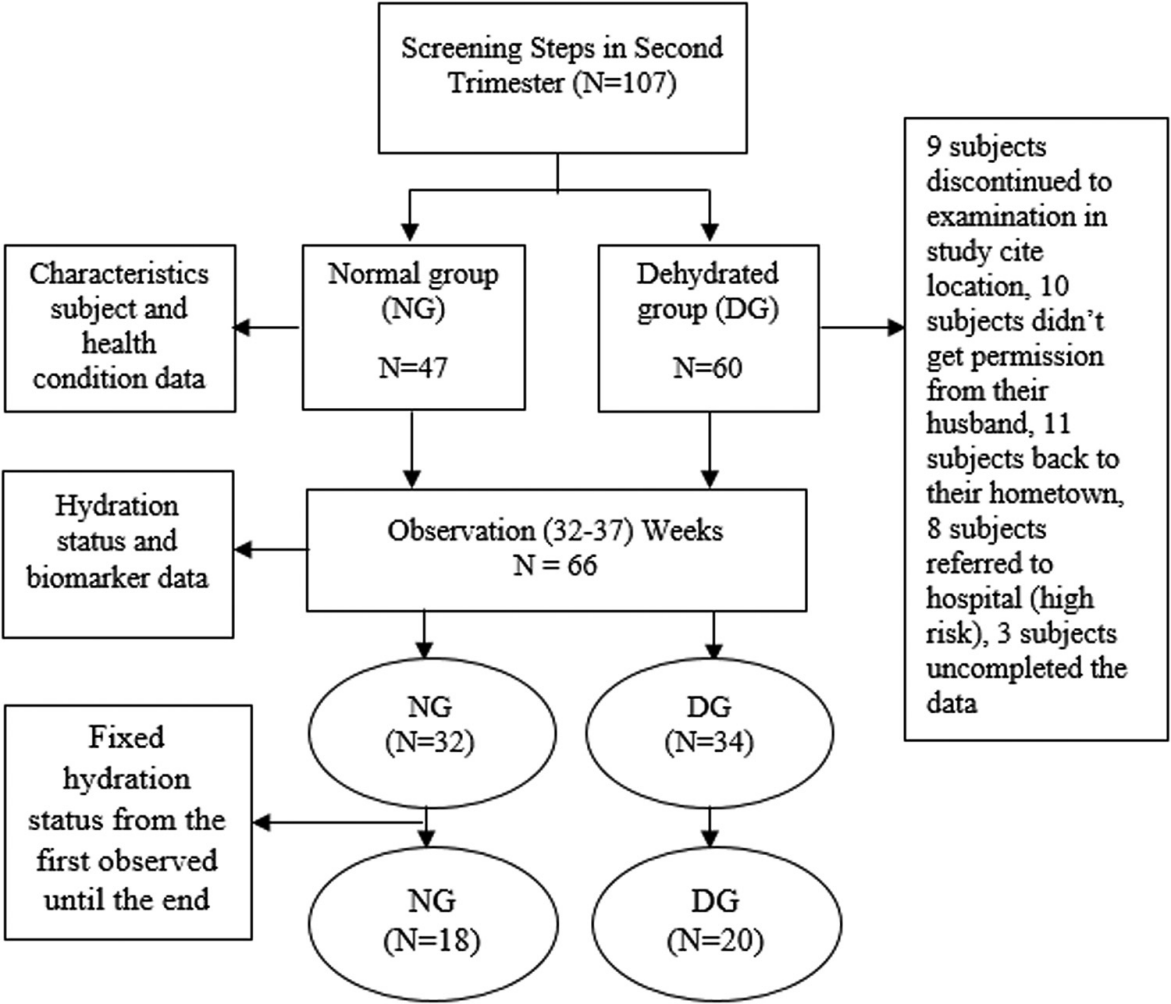
Flowchart Sampling.

Research subjects were second-trimester pregnant women (16–18 weeks’ gestation) who took part in the study until the end (parturition). The inclusion criteria were as follows; (1) doing pregnancy examination in the study site, (2) in the second and third trimester, (3) in normal health (no secondary infection) based on the medical record, (4) never had low-birth-weight or stunted (<48 cm), (5) aged between 18–35 years, (6) the height between 150–165 cm, (7) BMI (body mass index) of 18⋅5–25⋅0, (8) experienced urinary tract infection, (9) experienced vomiting, nausea, and diarrhea in the first trimester, (10) planned to delivery in the study site, (11) signed the informed consent, (12) willing to participate, and (13) never had a cesarean delivery. A total of 38 subjects with a fixed hydration status were observed until the end of the study, the flowchart of the subject sampling is presented on Figure 1. Subjects with fixed hydration status have the same hydration category after the observation period from 32–37 weeks. For instance, when a subject was dehydrated from 32 until 37 weeks. The subjects were categorized into the dehydration group (DG) (*n* 20) and the normal group (NG) (*n* 18).

### Data collecting

Subject characteristics include maternal age, weight before and during pregnancy, height, upper arm circumference, waist circumference, hip circumference, nutritional status before pregnancy, body temperature, pulse, fundus height, blood pressure, and hydration status. Characteristics were obtained through interviews and questionnaires completed by trained enumerators for 15–20 min. Then anthropometric measurements were performed by enumerators and midwives for 15–20 min. Measurements were done three times (twice by an enumerator and once by a midwife as a measurement data control). Meanwhile, data on nutritional status before pregnancy, blood pressure, and body temperature were obtained from the mother's medical record through the KIA (Kartu Ibu dan Anak) book (Maternal and Child Health Book).

Biomarker hydration status data used were urine color, urine specific gravity, serum sodium, urine, and serum osmolality measured from six observation time points. Urine and blood collection to measure hydration status at six points was carried out in the third trimester, namely the first three points each in the 32–34 week period and the second three points in the 35–37 week period. This collection method was to reduce the risk of pain when taking blood, hence frequented weekly. Then, look for the subjects who have a constant hydration status from the beginning to the end of the study. Biomarkers measurement of hydration status were taken through blood and urine. Urine-specific gravity test using the urinometer method, urine and plasma osmolality are checked through an osmometer, urine color checked through the PURI indicator, and serum sodium is examined through the ISE method. Table 1 shows the threshold level for each biomarker used to assess the hydration status.

**Table 1.** Threshold level of hydration biomarker

Biomarker indicator	Threshold
Serum Sodium^(17)^	1. 135–145 mEq/L (Well-hydrated)
	2. >145 mEq/L (Dehydrated)
Urine Specific Gravity (USG)^(14)^	1. 1⋅021–1⋅026 (Slightly dehydrated/Hypohydration)
	2. >1⋅026 (Chronic dehydration)
Urine Colour^(18)^	1. Score of 1–3 (Well-hydrated)
	2. Score of 4–6 (Slightly dehydrated)
	3. Score of 7–8 (Dehydrated)
Urine Osmolality^(18)^	1. <500 mOsm/kg (Normal)
	2. ≥500 mOsm/kg (Dehydrated)
Serum/Plasma Osmolality^(19)^	1. ≤299 mOsm/kg (Normal)
	2. >299 mOsm/kg (Dehydrated)

Hydration status data is obtained from five biomarkers average. If more than two indicators presented higher than normal, i.e., (a) ≥2 indicators: ≥60 % dehydration; (b). <2 indicators: <60 % non-dehydrated (normal). Biomarker data collection was assisted by phlebotomy personnel, while data on birth weight and length were obtained from the midwife measurements 30 min after birth.

Nutrition and water intake data were collected through 1 × 24 h recall interviews for six days, which were conducted three days (two working days, one day off) between 32–34 weeks of gestation and the following three days in between 35–37 weeks of gestation. Before observation, subjects were given an explanation of blood and urine collection and food and drink intake, where they were also encouraged to eat and drink as usual. The average intake for six days represents the subject intake during observations in the third trimester of pregnancy, and the use of 24-hour food recall was because it is more convenience for the subject, than giving them a 3 days food diary. The data was collected during blood and urine sampling to represent the nutrients and water intake in the third trimester of pregnancy and correct hydration status. Water level data in percentage is calculated based on water intake compared to the individual water needs based on RDA (Recommended Dietary Allowance). The previous study shows that <90 % is in the deficient category, and ≥90 % is sufficient^(14)^. Water intake data are obtained from: drinking water, other drinks (colored and flavored), water in food, and metabolic water.

Materials used in this study were blood (20 ml) and urine (50 ml) samples. Blood samples taken are intravenous on-the-spot blood samples. Urine taken is spot urine taken at 14⋅01–16⋅00 local time. Examinations related to hydration biomarkers: urine color, urine specific gravity, urine osmolality, plasma osmolality, and serum sodium are carried out by an Accredited Laboratory in collaboration with this study.

Equipment used in conducting the research included digital baby scales ‘Laica’ and meter line to measure body weight and height, fundus, upper arm, waist, and hip circumference, Combur 10 M (Roche) to measure urine specific gravity, Osmomat 3000 Gonotec GmBH to measure urine and serum osmolality, PURI Indicator to measure urine color, ADVIA 1800 (for sodium examination) with ADVIA Chemistry Ise Buffer reagents (Siemens).

### Data analysis

Data were processed and analyzed with Microsoft Office Excel and SPSS 20.0 software. Biomarkers of hydration status, subject characteristics, and birth output (birth weight and length) were analyzed descriptively and presented in the form of mean (mean), median, and standard deviation (sd). The bivariate analysis testing used a different t-two independent test to look at differences in subject characteristics, analysis of nutrient and water intake, and birth output on hydration status. ANCOVA test was conducted to evaluate birth output data on confounding variables. In this study, the confounding variable is water intake level that gives a possible-to-false association. Data were analyzed at a 95 % confidence level and significance level *P* < 0⋅05.

## Results

### Subjects characteristics based on hydration status

This study observed hydration status at the beginning and the end of the study. Of the total of 66 subjects being taken, 38 subjects had a fixed hydration status from the beginning until the end of observation. It was found that most of the subjects experienced dehydration during pregnancy (52⋅6 %). Based on Table 1, there were no differences in subject characteristics of the two groups of mothers, both dehydrated and normal (*P* ≥ 0⋅05), indicating the similarity of subject characteristics.

### Differences between nutritional intake and water intake based on hydration status during pregnancy

Table 2 shows no differences in energy, carbohydrate, protein, fat, iron, zinc, and calcium intake (*P* ≥ 0⋅05). However, there were differences in water intake levels in the two DG and NG (*P* < 0⋅05).

**Table 2.** Subject characteristics on the beginning of study (baseline)

Variable	DG, *n* 20	NG, *n* 18	*P*-value
Age (year)	26⋅1 (21⋅4–30⋅8)	26⋅9 (21⋅4–32.)4	0⋅63
Height (cm)	153⋅6 (148⋅7–158⋅5)	154⋅6 (149⋅3–159⋅9)	0⋅56
Gestational Age (week)	17⋅0 (16⋅1–17⋅9)	16⋅9 (16–17⋅8)	0⋅74
Weight on Second Trimester (kg)	61⋅5 (51⋅9–71⋅1)	56⋅7 (48⋅6–64.)8	0⋅10
Weight on Third Trimester (kg)	70⋅8 (59⋅8–81⋅8)	65⋅1 (55⋅9–74⋅3)	0⋅09
Maternal Weight Gain (kg)	12. (8⋅2–16⋅8)	11⋅7 (7⋅8–15⋅6)	0⋅54
Pregnancy Duration (Week)	38⋅7 (37⋅8–39⋅5)	38⋅5 (37⋅75–39⋅25)	0⋅50
Upper Arm Circumference (cm)	28⋅3 (25⋅2–31⋅4)	27⋅1 (24⋅4–29⋅8)	0⋅21
Fundus Height (cm)	13⋅7 (10⋅8–16⋅6)	13⋅6 (11⋅1–16⋅1)	0⋅87
Waist Circumference (cm)	91⋅5 (82⋅2–100⋅8)	88⋅7 (82⋅6–94⋅8)	0⋅27
Hip Circumference (cm)	101⋅0 (92⋅3–109⋅7)	91⋅9 (70⋅2–113⋅6)	0⋅09
Body Temperature (^0^C)	36⋅3 (35⋅6–37⋅0)	35⋅9 (33⋅6–38⋅2)	0⋅46
Pulse (bpm)	88⋅5 (68⋅7–108⋅3)	92⋅8 (72⋅6–113)	0⋅51
Blood Pressure:			
Systolic (mmHg)	110⋅8 (102⋅1–119⋅5)	109⋅4 (102⋅2–116⋅6)	0⋅60
Diastolic (mmHg)	68⋅3 (63⋅0–73⋅6)	69⋅7 (63⋅1–76⋅3)	0⋅48

Data were distributed normally.

DG, Dehydrated group; NG, Normal group.

Independent *t*-test, significant with *P* < 0⋅05.

The level of water intake is calculated based on the amount of percentage obtained from regular water, colored and flavored drinks, water in food, and metabolic water compared to water needs. This study found differences in water intake levels in the two groups. Based on the percentage value of water intake, the dehydrated mother's group is in the poor category and normal mothers are in the sufficient category.

### Dehydration effect on birth output

Table 3 shows that there were differences in birth output (birth weight and length, head circumference, and chest circumference) between the two groups (*P* < 0⋅05). Also, the table shows the effect of dehydration on the weight and length of the baby's body (*P* < 0⋅05). In dehydrated mothers, the weight, height, head circumference, and chest circumference of the baby are lower than the normal mother group.

**Table 3.** Differences between nutritional intake and water intake based on hydration status during pregnancy

Variable		DG, *n* 20	NG, *n* 18	*P*-value
Energy	(kcal)	1691⋅0 (1326⋅0–2056⋅0)	1607⋅0 (1252⋅0–1962⋅0)	0⋅48
	(%)^a^	62⋅9 (49⋅3–76⋅5)	61⋅2 (47⋅2–75⋅2)	0⋅70
Carbohydrate	(g)	214⋅0 (157⋅0–271⋅0)	213⋅0 (148⋅0–278⋅0)	0⋅93
	(%)^a^	92⋅7 (87⋅8–97⋅6)	95⋅8 (82⋅1–109⋅5)	0⋅50
Protein	(g)	59⋅5 (43⋅8–75⋅2)	57⋅0 (42⋅5–71⋅5)	0⋅62
	(%)^a^	93⋅1 (85⋅1–101⋅1)	94⋅0 (84⋅5–103⋅5)	0⋅75
Fat	(g)	70⋅9 (48⋅3–93⋅5)	64⋅5 (46⋅5–82⋅5)	0⋅34
	(%)^a^	69⋅9 (53⋅6–86⋅2)	71⋅0 (61⋅5–80⋅5)	0⋅81
Iron	(mg)	10⋅7 (3⋅6–17⋅8)	12⋅3 (0⋅0–25⋅9)	0⋅67
Zinc	(mg)	6⋅7 (4⋅6–8⋅8)	6⋅7 (4⋅9–8⋅5)	0⋅89
Calcium	(mg)	315⋅0 (168⋅0–462⋅0)	338⋅0 (131⋅0–545⋅0)	0⋅68
Water Intake	(ml)	2212⋅8 (1321⋅1–3065⋅7)	3393⋅7 (2707⋅7–4315⋅5)	0⋅01*
	(%)^a^	72⋅2 (60⋅6–83⋅8)	114⋅5 (98⋅2–130⋅8)	0⋅01*

Data were distributed normally.

DG, Dehydrated group; NG, Normal group.

a Percentage compared to the needs.

* Independent *t*-test, significant with *p*<0⋅05.

Table 4 shows that the level of water intake is related to hydration status. Further assessment on the effect of hydration status on pregnancy outcomes requires testing the water intake level correction on hydration status, which impacts pregnancy outcomes. After correcting the water intake level, the difference in weight and length of newborns from the (DG) and NG were 500⋅6 g and 0⋅4 cm, respectively. Meanwhile, differences in head circumference and chest circumference of newborns from the DG and NG were 0⋅8 cm and 1⋅4 cm, respectively (Showed in Table 4).

**Table 4.** Birth output differences based on hydration status

Variable	DG, *n* 20	NG, *n* 18	*P*-value
Birth Weight (kg)	2862⋅3 (2542⋅7–3181⋅3)	3281⋅9 (2914⋅2–3649⋅6)	0⋅01*
Birth Length (cm)	48⋅2 (47⋅2–49⋅2)	48⋅8 (48⋅0–49⋅6)	0⋅03*
Head Circumference (cm)	32⋅3 (31⋅4–33⋅2)	33⋅4 (32⋅7–34⋅1)	0⋅01*
Chest Circumference (cm)	32⋅3 (31⋅2–33⋅4)	33⋅7 (32⋅9–34⋅5)	0⋅01*
Pulse (bpm)	141⋅1 (139⋅6–142⋅6)	140⋅7 (138⋅3–143⋅1)	0⋅50
Placenta Weight (g)	640⋅4 (588⋅6–692⋅2)	629⋅3 (582⋅8–675⋅8)	0⋅49

Data were distributed normally.

DG, Dehydrated group; NG, Normal group.

* Independent *t*-test, significant with *P* < 0⋅05.

Table 5 also explains that the difference in the value of the correction of water intake to the birth length of DG and NG were 0⋅1 cm and −0⋅1 cm, respectively. As for the bodyweight of the DG and NG, respectively −38⋅3 g and 42⋅6 g. This is similar to the head circumference in the group of dehydrated (normal) and normal (NG) mothers, namely 0⋅1 cm and −0⋅2 cm. Chest circumference in the DG and NG maternal groups were 0⋅0 cm and 0⋅0 cm, respectively.

**Table 5.** Dehydration effect on birth output

Variable	DG, *n* 20	NG, *n* 18
Birth Weight (g)*		
Before correction	2862⋅2 (2542⋅9–3181⋅5)	3281⋅9 (2914⋅2–3649⋅6)
After correction	2823⋅9 (2710⋅7–2937⋅1)	3324⋅5 (3201⋅7–3447⋅3)
Birth Length (cm)*		
After correction	48⋅2 (47⋅2–49⋅2)	48⋅8 (48–49⋅6)
Before correction	48⋅3 (48⋅0–48⋅6)	48⋅7 (48⋅4–49⋅0)
Head Circumference (cm)*		
Before correction	32⋅3 (31⋅4–33⋅2)	33⋅4 (32⋅7–34⋅1)
After correction	32⋅4 (32⋅2–32⋅6)	33⋅2 (32⋅9–33⋅5)
Chest Circumference (cm)*		
Before correction	32⋅3 (31⋅2–33⋅4)	33⋅7 (33–34⋅4)
After correction	32⋅3 (32⋅0–32⋅6)	33⋅7 (33⋅4–34⋅0)

DG, Dehydrated group; NG. Normal group.

* ANCOVA, significant with *P* < 0⋅05. R-squared: birth weight 0⋅28, birth length 0⋅11, head circumference 0⋅32, chest circumference 0⋅34. Before and after corrected with water intake level.

## Discussion

The maintenance of fluid and electrolyte balance is essential for a healthy quality of life during pregnancy. Dehydration, overhydration, salt balance, and excess water are associated with morbidity and death, especially in the elderly, in which the risk is increasing^(20)^. This study is in line with preliminary research, which states that 57⋅1 % of pregnant women experience dehydration^(21)^.

Medical prevalence in pregnancy cases is increasing because of the complex interactions between demographic and lifestyle factors and modern medical science development. Two-thirds of maternal deaths from 2011 to 2013 occurred in women with known medical comorbidities, and 30 % of women who died were obese^(22)^. Thus, pregnancy health problems are an important concern in the case of maternal and child health.

This study showed that maternal weight gain from both groups DG and NG was normal. A consistent weight gain in the pregnancy period was associated with better pregnancy output. Excessive weight gain can affect pregnancy output and, in the short term, will be very dangerous. Therefore, some studies necessitated planning and monitoring weight gain during pregnancy^(23, 24)^.

Weight gain is influenced by socio-demographic, lifestyle, and genetic factors. These risk factors will increase the risk of the adverse event in the mother, fetus, and child^(25)^. Thus, anthropometric examination (with weight monitoring) needs to be done.

Epidemiological studies showed a correlation between food intake and health, including water intake to determine health status and weight loss in overweight women^(26, 27)^. This study demonstrated a relationship between water intake level and hydration status. Dehydration has a serious consequence for older adults, including an increased risk of illness or death. Chronic dehydration occurs for a long time, which happens in older adults 48 %^(28)^.

During normal pregnancy conditions, an increase in plasma volume is needed for the body balance mechanism^(29)^. Thirst is vital in maintaining body fluid homeostasis and avoiding deficiency in intracellular and extracellular fluid volumes. Thirst occurs because nerve signals arising from osmotic and hormonal influences on the terminal lamina can be integrated into the brain, with afferent information transmitted from intrathoracic baroreceptors through the hindbrain to stimulate thirst^(30, 31)^.

Water intake consists of about 20 % of the water from solid food and 80 % water from drinks and drinking water^(32)^. Water intake is important for maintaining one's hydration status, one of which can be demonstrated by the value of urine osmolality^(18, 33)^. Thus, in the case of pregnancy, it is important to maintain the intake of nutrients and especially water to prevent pregnancy problems.

Dehydration is a predictor of increased fragility, deteriorating mental performance, and quality of life^(34)^. This study explains that the incidence of chronic dehydration during pregnancy impacts the birth weight and length also on the head and chest circumference of the baby. According to Tanner (1978), the speed of weight growth starts at 32 weeks gestation and peak at 34 weeks gestation. At 34–36 weeks gestation, fetal growth slows down because the uterus narrowing^(35)^, but significantly increased in the first six months postpartum, especially in the first eight weeks after the baby is born. At this age, mothers tend to feel full, so they limit their water intake even though it is a fact that an adequate intake of nutrients and water is needed to support fetal growth during this period.

Mild dehydration occurred when 2 % weight loss happened, which caused headaches, fatigue and reduces physical and mental performance^(36)^. Chronic dehydration repeatedly occurs and for a long time^(37)^. Therefore, it is necessary to consider the intake of nutrients and water in pregnant women.

Feeding during pregnancy and nutrient supply from mother to fetus is needed to develop embryos/fetus and fetal growth, cell proliferation, and DNA replication in critical periods^(38)^. Another study in rats also found the same effect of dehydration on birth weight and length^(39)^. In addition, lack of water intake can cause a lack of amniotic fluid in pregnant women, which can cause oligohydramnios. So, in this case, the mother needs intravenous fluid management^(40)^. In line with other studies, the disruption of sodium balance in pregnant rats interferes with hydration status and impacts pregnancy output^(41, 42)^.

It is known that head circumference in infants and children is an essential indicator of growth and development. In another study found that the baby's head circumference was positively correlated with brain volume. Low head circumference is at risk for low brain volume^(43)^. In addition, other studies have also shown that head circumference is associated with stunting and gross measures of neurological development^(44)^.

Thus, more attention to pregnant women's nutrients and water intake is necessary to support fetal growth and development. Water has a critical role in maintaining the body's metabolic activity, its contribution through cell volume homeostasis. Changes in cell volume have important implications for regulating the intake of nutrients and metabolic waste and affect cell metabolism and gene expression^(35, 38, 42)^.

This study was conducted during pregnancy, starting from the screening period in the first trimester to observation in the second, third trimester, and at birth. The small number of subjects due to exclusion became one of this study's limitations. In addition, the study site only represents areas with lower-middle economic characteristics. This study did not measure the physical activity of pregnant women that can correct hydration status and oxidative stress. So, the possibility of other factors such as physical activity, access to food and fluid, clinical testing of the baby's condition can provide an overview of the impact of dehydration.

## Conclusion

A total of 52⋅6 % of subjects experienced dehydration during the pregnancy period. There are differences in water intake levels between dehydrated and normal mothers. There were differences in body weight and length, head circumference, and chest circumference of the newborns among two groups of pregnant women. There was a positive relationship between hydration status and pregnancy outcome, indicated by the difference in birth weight and length, head and chest circumference between two groups: 500⋅6 g and 0⋅4 cm, 0⋅8 cm and 1⋅4 cm, respectively.

It is recommended that mothers during pregnancy monitor their health by routinely carrying out the weight and a simple hydration status check and maintaining a pattern of intake of food and drink by drinking 3⋅0 l per day. Further research requires more subjects to observe the effects of chronic maternal dehydration on pregnancy output using a cohort design that longitudinally monitor the first six months of life. According to the theory, six months of observation is necessary, suggesting the first six months, especially the age of 8 weeks, as the golden age for catching up, especially weight in babies^(45)^.
